# High-Yield Production of Levulinic Acid from Pretreated Cow Dung in Dilute Acid Aqueous Solution

**DOI:** 10.3390/molecules22020285

**Published:** 2017-02-14

**Authors:** Jialei Su, Feng Shen, Mo Qiu, Xinhua Qi

**Affiliations:** Agro-Environmental Protection Institute, Chinese Academy of Agricultural Sciences, No. 31, Fukang Road, Nankai District, Tianjin 300191, China; sujialei7865768@126.com (J.S.); shenwindy@126.com (F.S.); qiumo@aepi.org.cn (M.Q.)

**Keywords:** biomass, agricultural residue, value-added chemical, glucose, levulinic acid, biorefinery

## Abstract

Agricultural waste cow dung was used as feedstock for the production of a high value–added chemical levulinic acid (LA) in dilute acid aqueous solutions. A high LA yield of 338.9 g/kg was obtained from the pretreated cow dung, which was much higher than that obtained from the crude cow dung (135 g/kg), mainly attributed to the breakage of the lignin fraction in the lignocellulose structure of the cow dung by potassium hydroxide (KOH) pretreatment, and thus enhanced the accessibility of cow dung to the acid sites in the catalytic reaction. Meanwhile, another value-added chemical formic acid could be obtained with a yield of ca. 160 g/kg in the process, implying a total production of ca. 500 g/kg yield for LA and formic acid from the pretreated cow dung with the proposed process. The developed process was shown to be tolerant to high initial substrate loading with a satisfied LA yield. This work provides a promising strategy for the value-increment utilization of liglocellulosic agricultural residues.

## 1. Introduction

Lignocellulosic biomass is the most abundant biomass in the world, which can be converted into various valuable platform compounds such as furfural, lactic acid, formic acid and levulinic acid by biorefinery processes [1,2,3,4,5]. Among these compounds, levulinic acid (LA) is a short-chain fatty acid with a ketone carbonyl and an acidic carboxyl group, which has wide applications in industry and agriculture to produce a variety of products such as plasticizer, oil additives and fragrances [6,7,8,9]. Up to now, the production of LA has been broadly investigated from a variety of feedstocks such as sugars, cellulose, chitin and raw lignocellulosic biomasses by homogeneous or heterogeneous catalysts [4,5,9,10,11,12]. Compared with homogeneous mineral acid catalysts such as HCl, H_2_SO_4_ and H_3_PO_4_, heterogeneous catalysts such as acidic ion-exchange resins, heteropoly acids and metal oxides can be easily isolated from the reaction mixture and reused, and they have no corrosion problem. However, heterogeneous catalysts are still not satisfactory in LA production, especially from cellulose and raw biomasses feedstocks, due to the solid-solid mass transfer limitation [13,14,15]. Therefore, although mineral acids have the disadvantages of equipment corrosion and difficulty in recycling, they have been broadly used in producing LA and are beneficial for industrial-scale production because of their high activity and low cost [16,17,18]. In addition, the corrosion problem of the equipment can be partially avoided by the employment of dilute acid or acid-resistant materials [15].

**Scheme 1:**
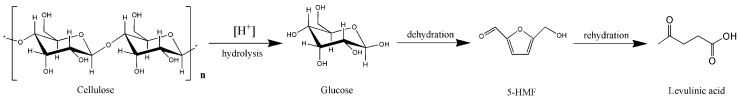
Reaction pathway for the acid-catalyzed conversion of cellulose to levulinic acid.

In terms of starting materials used for LA production, although monosaccharides such as glucose and fructose can afford high LA yields [15,19], the high cost of sugars restricts their application in LA production. Therefore, the production of LA from cellulose and, in particular, from raw and waste biomasses is preferred due to their abundance and low cost. When cellulose or raw lignocellulosic biomasses are used as starting materials, LA is usually produced by the acid-catalyzed conversion of cellulose, following a reaction pathway in which cellulose is hydrolyzed into glucose via enzymatic or acid-catalysis methods, and the generated glucose is dehydrated to 5-hydroxymethylfurfural (HMF), followed by a rehydration step converting HMF to LA, as shown in Scheme 1 [15,17,20,21,22].

In the past decades, the increasing demand for beef and milk has dramatically promoted the development of the cattle industry and led to the production of a great amount of cow dung waste [23]. At present, the primary use of cow dung is land-applied fertilizer. However, excess land application of cow dung results in some serious environmental problems, such as high excess loading of nitrogen and phosphorus and groundwater pollution [24,25]. Therefore, cow dung management is becoming an environmental challenge [26]. Although cow dung is currently utilized by means of compost, pyrolysis, combustion and anaerobic co-digestion [27,28,29], more efforts are needed to develop new cow dung management strategies.

Cow dung is the waste product excreted from bovine animal species that use agricultural residues such as grass and rice straw as food. From the aspect of chemical composition, cow dung is classified as a lignocellulosic biomass resource which mainly consists of cellulose, hemicellulose and lignin. Thus, it is a potential feedstock for the production of value-added chemicals. In this work, an attempt on the conversion of cow dung into the valuable chemical levulinic acid was made, where cow dung was first pretreated with KOH solution to destroy the recalcitrance of the lignocellulose structure and increase its accessibility, and then levulinic acid was produced from the pretreated cow dung by diluted acid, and a high LA yield of 338.9 g/kg was obtained. This work provides a promising strategy for the value-increment utilization of agricultural residue cow dung.

## 2. Results and Discussion

### 2.1. Morphology Change of Cow Dung before and after Pretreatment

**Figure 1:**
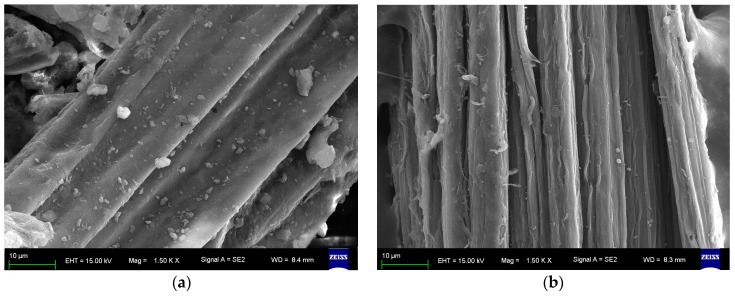
SEM image of (**a**) crude cow dung and (**b**) pretreated cow dung.

The morphology of the cow dung before and after pretreatment was observed using a scanning electron microscope (Merlin, Germany) (Figure 1). It can be seen that the crude cow dung had an intact and smooth surface (Figure 1a). After pretreatment, the fiber became rougher and thinner. The fiber surface seemed to be eroded and ruptured and some parts of the fibers were broken into separate fibers. The morphological alteration after pretreatment was due to the extensive removal of lignin which provides cohesion between cellulose microfibrils. Theses alterations would promote cellulose hydrolysis by exposing the active site of the cellulose and increasing the accessible specific surface area of the cellulose fraction.

### 2.2. Catalytic Production of Levulinic Acid from Pretreated Cow Dung by Different Liquid Acids

**Figure 2:**
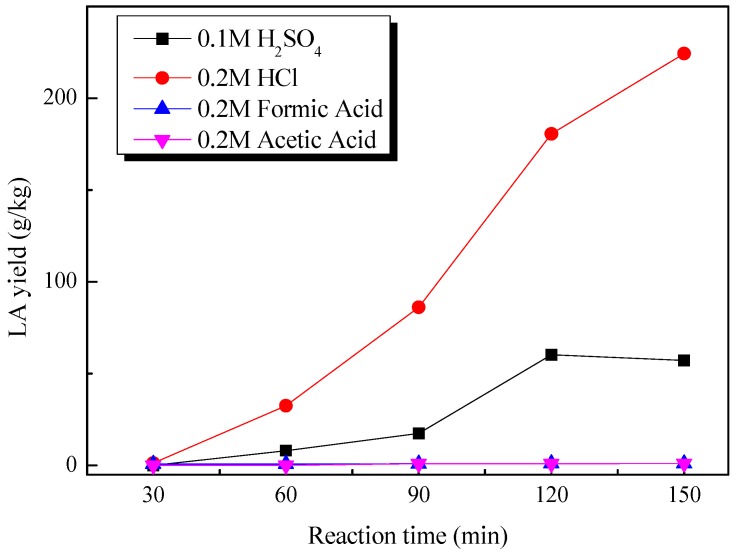
Production of LA from pretreated cow dung catalyzed by different liquid acid catalysts (reaction conditions: 0.3 g pretreated cow dung, acid aqueous solution 10 mL, 180 °C).

As the most common acid catalysts, liquid acids such as hydrochloric acid, sulfuric acid and phosphoric acid are widely used in lignocellulosic biomass conversion because of their effectiveness and convenience [30,31]. The production of levulinic acid from the pretreated cow dung as a starting material was examined with different liquid acids, including two mineral acids (H_2_SO_4_ and HCl) and two organic acids (formic acid and acetic acid). The results are illustrated in Figure 2. It can be seen that formic acid and acetic acid almost had no catalytic activity for the conversion of the pretreated cow dung into levulinic acid under the reaction conditions, due to their weak acidity. When H_2_SO_4_ was used, a LA yield of 60.3 g/kg was obtained at 180 °C in a 120 min reaction time, accompanied by other products, including glucose (215.8 g/kg), 5-hydroxymethylfurfural (9.1 g/kg), and formic acid (33.2 g/kg). Among these acids, HCl exhibited the highest activity for the production of LA from the pretreated cow dung, and a high, unoptimized LA yield of 225 g/kg could be achieved at 180 °C for 150 min. The high activity of HCl should be ascribed to the fact that the chloride ion can combine with the hydroxyl group in the cellulose molecule to destroy the hydrogen bonds within the cellulose molecule and thus promote the hydrolysis of cellulose [32,33].

### 2.3. Catalytic Production of LA from Crude and Pretreated Cow Dung in HCl Aqueous Solutions

**Figure 3:**
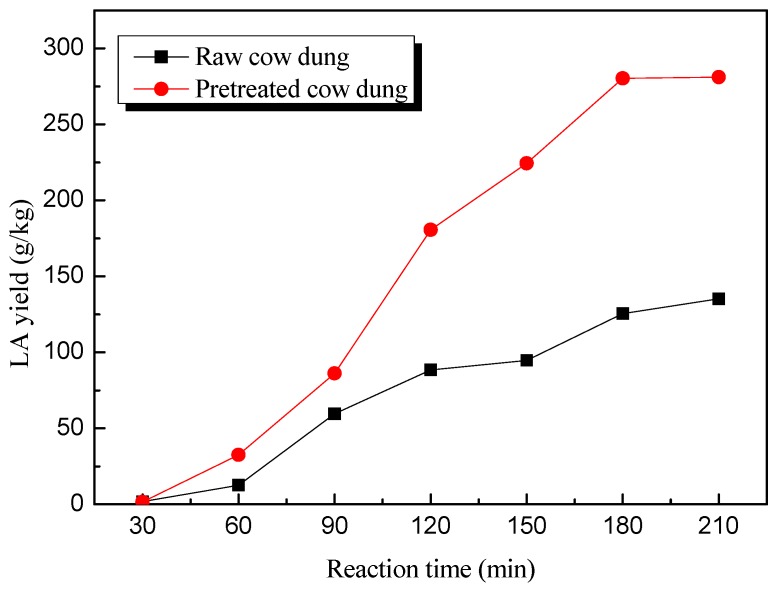
Catalytic production of LA from cow dung with and without pretreatment in HCl aqueous solutions (reaction conditions: 0.3 g substrate, 0.2 M HCl aqueous solution 10 mL, 180 °C).

Since HCl showed the highest catalytic activity for the conversion of cow dung into LA, HCl was used as the catalyst in the following work. To explore the effect of pretreatment of the cow dung on the LA production, the production of LA from cow dung with and without pretreatment was examined at 180 °C in 0.2 M HCl aqueous solution (Figure 3). It can be seen that the crude cow dung could also be converted to LA, and a LA yield of 135 g/kg was obtained in a 210 min reaction time. However, the pretreated cow dung afforded a high LA yield of 280 g/kg in 180 min, which was much higher than that obtained from the cow dung without pretreatment. The results imply that the pretreatment for the cow dung plays a vital role in converting the cow dung into LA, which should be attributed to the removal of the lignin fraction from the lignocellulose structure of the cow dung by the KOH solution in the pretreatment step, improving the accessibility of the cow dung to the acid sites in the catalytic reaction.

### 2.4. Effect of the Reaction Temperature on the Production of LA from Pretreated Cow Dung

**Figure 4:**
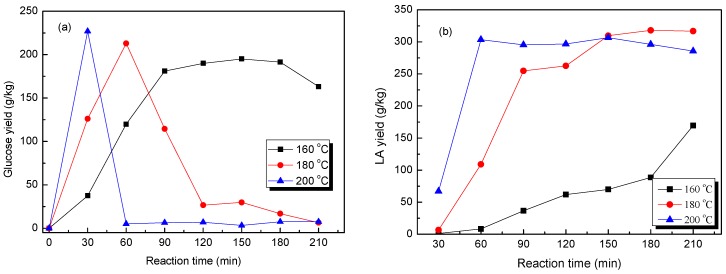
Effect of reaction temperature on the production of (**a**) glucose and (**b**) LA from cow dung. (reaction conditions: 0.3 g pretreated cow dung, 0.4 M HCl aqueous solution 10 mL).

In the catalytic conversion of lignocellulosic biomass, the reaction temperature is an important effect factor that plays a crucial role in determining the products’ distribution [6,34]. LA is generally produced by the rehydration of 5-hydroxymethylfurfural [35], which is obtained by the dehydration of glucose or fructose through eliminating three water molecules [16]. The hydrolysis of cellulose to glucose is considered to be a key step in the production of LA directly from lignocellulosic biomass [36,37]. Generally, compared with the hydrolysis of cellulose, the production of levulinic acid needs higher temperatures [10,38,39]. The influence of the reaction temperature on the production of LA from pretreated cow dung was investigated (Figure 4). When the reaction was conducted at 160 °C, the LA yield increased slowly with the increasing reaction time, and a LA yield of 169.7 g/kg was attained in a 210 min reaction time. In this case, glucose was the main product that reached around 190 g/kg yield within 90 min. When the temperature was increased to 180 °C and 200 °C, the glucose yields first reached peak values of 213.0 and 226.9 g/kg within 60 and 30 min, respectively, and then rapidly decreased, accompanied by rapid increases of the LA yield to 309.7 and 306.6 g/kg in a 150 min reaction time, respectively. Thus, 180 °C was an optimum temperature for the production of LA from cow dung in the process and was adopted in the following work.

### 2.5. Effect of Loading Substrate Amount on the Production of LA from Cow Dung

**Figure 5:**
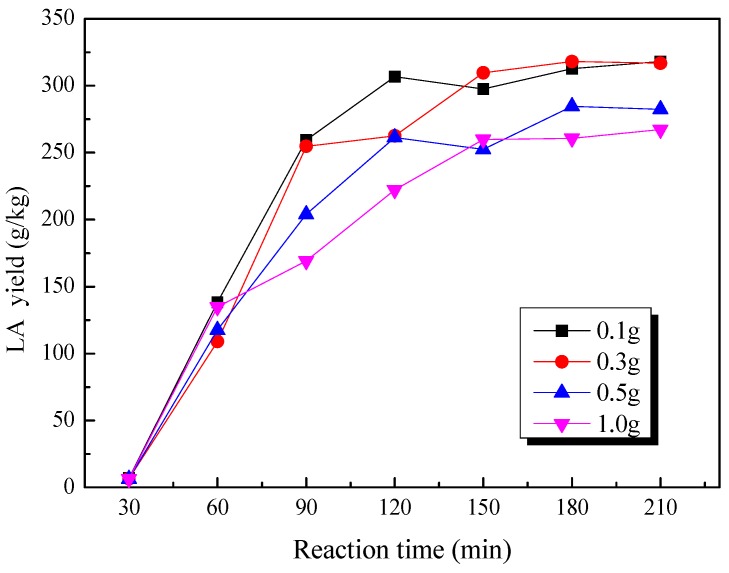
Effect of substrate loading amount on LA production from cow dung (reaction conditions: 0.4 M HCl aqueous solution 10 mL, 180 °C).

The influence of the loading substrate amount on the production of LA from the pretreated cow dung was examined, and the results are shown in Figure 5. The experiments were carried out in 0.4 M HCl aqueous solution at 180 °C, and the loading amounts of the pretreated cow dung were 0.1, 0.3, 0.5 and 1.0 g. When relatively low concentrations of pretreated cow dung (0.01 and 0.03 g) were applied in the reaction system, maximum LA yields of ca. 315 g/kg were obtained in a 180 min reaction time. When the loading amount of the pretreated cow dung was further increased to 0.5 and 1.0 g, the decreases in the LA yield were limited, and LA yields of 284.7 and 260.7 g/kg were obtained in a 180 min reaction time, respectively. The results indicate that the initial loading substrate concentration had little effect on the LA yields from the pretreated cow dung in the reaction system, and the proposed catalytic system is applicable for the production of LA from the pretreated cow dung with high initial substrate loading up to 10 wt. %, which should be favorable for practical engineering applications.

### 2.6. Effect of Acid Concentration on the Production of LA from Cow Dung

**Figure 6:**
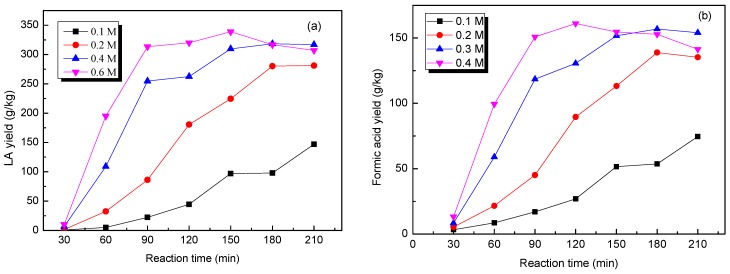
Effect of the acid concentration in HCl aqueous solutions on the production of (**a**) LA and (**b**) formic acid (reaction conditions: 0.3 g pretreated cow dung, HCl aqueous solution 10 mL, 180 °C).

The effect of the acid concentration in the HCl aqueous solution on the catalytic production of LA from the pretreated cow dung was investigated (Figure 6). When the reaction was performed in 0.1 M HCl aqueous solution, the LA yield increased slowly with the prolonged reaction time, and a LA yield of 146.9 g/kg was achieved at 180 °C in a 210 min reaction time. With increasing the HCl concentration in the aqueous solutions, the LA yield significantly increased, and maximum LA yields of 309.7 and 338.9 g/kg were obtained in 150 min in 0.4 and 0.6 M HCl aqueous solutions, respectively, corresponding to LA yields of 73.6% and 80.5% based on the LA theoretical maximum yield, respectively. It should be noticed that another value-added chemical, formic acid, was produced in the reaction, which had a similar tendency as LA during reactions under different conditions. A high formic acid yield of ca. 160 g/kg could be achieved in 0.4 and 0.6 M HCl aqueous solutions, corresponding to a formic acid yield of ca. 95.6% based on the theoretical max yield of the formic acid. This indicated that a total yield of ca. 500 g/kg could be obtained for LA and formic acid from the pretreated cow dung in the proposed reaction system.

## 3. Experimental Methods

### 3.1. Materials

Formic acid, acetic acid, sulfuric acid, hydrochloric acid and potassium hydroxide were purchased from Sinopharm Chemical Reagent Co., Ltd. (Beijing, China). Cow dung was taken from a local cattle farm in Tianjin (China). The cow dung was dried at 105 °C for 24 h and was then ground to pass through a 40 mesh sieve before use.

### 3.2. Pretreatment of Cow Dung

Pretreatment of the cow dung was conducted with KOH aqueous solution to remove lignin component, as the following procedure. The cow dung was immersed into a 6% KOH aqueous solution with a solid–liquid ratio of 1:10, which was then loaded into a 200 mL Teflon lined stainless steel autoclave. The autoclave was heated to 120 °C and kept for 4 h in an oven. After that, the autoclave was cooled and the black liquor mainly consisting of lignin and KOH was separated from the mixture by centrifugation, and can be used to prepare porous carbon materials for catalysts and supercapacitors, following the procedure in our previous work [40]. The collected residual solid material was subsequently washed repeatedly with ultra-pure water till the filtrate became neutral, following with drying at 80 °C for 24 h. The main composition of the cow dung before and after pretreatment by KOH aqueous solution was determined by the National Renewable Energy Laboratory (NREL) method [41], and the results are listed in Table 1.

### 3.3. Catalytic Treatment of Cow Dung and Products Analysis

The catalytic reaction was carried out in a 50 mL Teflon lined stainless steel autoclave. Typically, 0.3 g pretreated cow dung was placed in 10 mL of 0.2 M HCl aqueous solution and loaded into the reactor. Then the closed reactor was magnetically stirred at 1000 r/min and heated to 180 °C and kept for the given reaction time. After reaction, the reactor was quenched with cold water and the obtained liquid sample after filtration was subjected to analysis with HPLC (Waters Acquity UPLC H-Class) equipped with RI detector and a Shodex SUGAR SH1011 column. The temperature of column and the RI detector was set to 50 and 35 °C, respectively. Dilute sulfuric acid aqueous solution (5 mM) was used as mobile phase at a flow rate of 0.5 mL/min.

The LA or formic acid yield based on the weight of the loaded cow dung was calculated using the following equation:

(1) $$\text{LA or formic acid yield (g/kg)}=\;\frac{\mathrm{mass}\;\mathrm{of}\;\mathrm{the}\;\mathrm{produced}\;\mathrm{LA}\;\mathrm{or}\;\mathrm{formic}\;\mathrm{acid}\;\mathrm{in}\;\mathrm{product}}{\mathrm{loading}\;\mathrm{mass}\;\mathrm{of}\;\mathrm{substrate}}$$

Theoretically, only cellulose can be transferred into levulinic acid in this work, and 1 mole glucose unit can produce 1 mole LA. According to the cellulose content in the pretreated cow dung (58.9%), theoretical max yield of LA was calculated to be 421 g/kg, based on the following equation: theoretical max yield of LA (g/kg) = cellulose content (g) × 0.716/loaded substrate weight (kg) [42]. Since 1 mole formic acid is produced for every mole of LA production from the rehydration of HMF, the theoretical max yield of formic acid (g/kg) from the pretreated cow dung was calculated to be 167.3 g/kg, according to the following equation: theoretical max yield of formic acid = (cellulose content (g) × 0.284/loaded substrate weight (kg).

All experiments were replicated at least three times and the average values based on the repeated trials were adopted in this work. The reproducibility showed a standard deviation within 3%.

## 4. Conclusions

In this work, lignocellulosic agricultural waste cow dung was used as a feedstock to produce the value-added chemical levulinic acid in dilute HCl aqueous solutions. Crude cow dung could afford a LA yield of 135 g/kg at 180 °C in a 210 min reaction time, and the LA yield could be improved to 338.9 g/kg after the cow dung was pretreated with KOH aqueous solution, ascribed to the enhancement of the accessibility of cow dung to the acid sites in the catalytic reaction, due to the removal of the lignin fraction from the lignocellulose structure of the cow dung by the KOH solution in the pretreatment step. In addition, a formic acid yield of ca. 160 g/kg could be achieved in the process, indicating that a total yield of ca. 500 g/kg could be obtained for LA and formic acid from the pretreated cow dung in the proposed reaction system. Compared with the conventional utilization method for cow dung, this work provides a promising strategy for the value-increment utilization of cow dung.

## Figures and Tables

**Table 1:** Main chemical components of the crude and pretreated cow dung.

Components	Crude Cow Dung	Pretreated Cow Dung
Cellulose, %	27.2	58.9
Hemicellulose, %	17.2	10.8
Lignin (acid insoluble), %	20.6	8.5
Ash, %	7.6	6.0

## References

[1] Yabushita M., Kobayashi H., Fukuoka A. (2014). Catalytic transformation of cellulose into platform chemicals. Appl. Catal. B-Environ..

[2] Elumalai S., Agarwal B., Sangwan R.S. (2016). Thermo-chemical pretreatment of rice straw for further processing for levulinic acid production. Bioresour. Technol..

[3] Ren J., Wang W., Yan Y., Deng A., Chen Q., Zhao L. (2016). Microwave-assisted hydrothermal treatment of corncob using tin(IV) chloride as catalyst for furfural production. Cellulose.

[4] Antonetti C., Bonari E., Licursi D., Di Nasso N.N.O., Galletti A.M.R. (2015). Hydrothermal Conversion of Giant Reed to Furfural and Levulinic Acid: Optimization of the Process under Microwave Irradiation and Investigation of Distinctive Agronomic Parameters. Molecules.

[5] Mukherjee A., Dumont M.-J., Raghavan V. (2015). Review: Sustainable production of hydroxymethylfurfural and levulinic acid: Challenges and opportunities. Biomass Bioenergy.

[6] Yuan Z., Long J., Xia Y., Zhang X., Wang T., Ma L. (2016). Production of Levulinic Acid from Pennisetum alopecuroides in the Presence of an Acid Catalyst. Bioresources.

[7] Gavilà L., Constantí M., Medina F. (2015). d-Lactic acid production from cellulose: Dilute acid treatment of cellulose assisted by microwave followed by microbial fermentation. Cellulose.

[8] Tabasso S., Grillo G., Carnaroglio D., Gaudino E.C., Cravotto G. (2016). Microwave-Assisted gamma-Valerolactone Production for Biomass Lignin Extraction: A Cascade Protocol. Molecules.

[9] Pileidis F.D., Titirici M.-M. (2016). Levulinic Acid Biorefineries: New Challenges for Efficient Utilization of Biomass. ChemSusChem.

[10] Sun Z., Xue L.F., Wang S.T., Wang X.H., Shi J.Y. (2016). Single step conversion of cellulose to levulinic acid using temperature-responsive dodeca-aluminotungstic acid catalysts. Green Chem..

[11] Flannelly T., Lopes M., Kupiainen L., Dooley S., Leahy J.J. (2016). Non-stoichiometric formation of formic and levulinic acids from the hydrolysis of biomass derived hexose carbohydrates. RSC Adv..

[12] Jeong G.T. (2014). Production of levulinic acid from glucosamine by dilute-acid catalyzed hydrothermal process. Ind. Crops Prod..

[13] Weingarten R., Conner W.C., Huber G.W. (2012). Production of levulinic acid from cellulose by hydrothermal decomposition combined with aqueous phase dehydration with a solid acid catalyst. Energy Environ. Sci..

[14] Yu F., Thomas J., Smet M., Dehaen W., Sels B.F. (2015). Molecular design of sulfonated hyperbranched poly(arylene oxindole)s for efficient cellulose conversion to levulinic acid. Green Chem..

[15] Antonetti C., Licursi D., Fulignati S., Valentini G., Raspolli Galletti A. (2016). New Frontiers in the Catalytic Synthesis of Levulinic Acid: From Sugars to Raw and Waste Biomass as Starting Feedstock. Catalysts.

[16] Fachri B.A., Abdilla R.M., van de Bovenkamp H.H., Rasrendra C.B., Heeres H.J. (2015). Experimental and Kinetic Modeling Studies on the Sulfuric Acid Catalyzed Conversion of d-Fructose to 5-Hydroxymethylfurfural and Levulinic Acid in Water. ACS Sustain. Chem. Eng..

[17] Bevilaqua D.B., Rambo M.K.D., Rizzetti T.M., Cardoso A.L., Martins A.F. (2013). Cleaner production: Levulinic acid from rice husks. J. Clean. Prod..

[18] Joshi S.S., Zodge A.D., Pandare K.V., Kulkarni B.D. (2014). Efficient conversion of cellulose to levulinic acid by hydrothermal treatment using zirconium dioxide as a recyclable solid acid catalyst. Ind. Eng. Chem. Res..

[19] Upare P.P., Yoon J.W., Kim M.Y., Kang H.Y., Hwang D.W., Hwang Y.K., Kung H.H., Chang J.S. (2013). Chemical conversion of biomass-derived hexose sugars to levulinic acid over sulfonic acid-functionalized graphene oxide catalysts. Green Chem..

[20] Elumalai S., Agarwal B., Runge T.M., Sangwan R.S. (2016). Integrated two-stage chemically processing of rice straw cellulose to butyl levulinate. Carbohydr. Poly..

[21] Yang Z.G., Kang H.Y., Guo Y.F., Zhuang G.Q., Bai Z.H., Zhang H.X., Feng C.X., Dong Y.P. (2013). Dilute-acid conversion of cotton straw to sugars and levulinic acid via 2-stage hydrolysis. Ind. Crops Prod..

[22] Peng L.C., Lin L., Zhang J.H., Zhuang J.P., Zhang B.X., Gong Y. (2010). Catalytic Conversion of Cellulose to Levulinic Acid by Metal Chlorides. Molecules.

[23] Xue B., Wang L.Z., Yan T. (2014). Methane emission inventories for enteric fermentation and manure management of yak, buffalo and dairy and beef cattle in China from 1988 to 2009. Agric. Ecosyst. Environ..

[24] Yang Q., Zhou S.F., Runge T. (2016). Dairy manure as a potential feedstock for cost-effective cellulosic bioethanol. Bioresources.

[25] Gollehon N., Caswell M., Ribaudo M., Kellogg B., Lander C., Letson D. (2001). Confined animal production and manure nutrients. Neuropharmacology.

[26] Stowe E.J., Coats E.R., Brinkman C.K. (2015). Dairy manure resource recovery utilizing two-stage anaerobic digestion—Implications of solids fractionation. Bioresour. Technol..

[27] Arikan O.A., Mulbry W., Rice C. (2016). The effect of composting on the persistence of four ionophores in dairy manure and poultry litter. Waste Manag..

[28] Wu H.J., Hanna M.A., Jones D.D. (2012). Thermogravimetric characterization of dairy manure as pyrolysis and combustion feedstocks. Waste Manag. Res..

[29] Song Z., Chao Z. (2015). Anaerobic codigestion of pretreated wheat straw with cattle manure and analysis of the microbial community. Bioresour. Technol..

[30] Liao W., Liu Y., Liu C., Wen Z., Chen S. (2006). Acid hydrolysis of fibers from dairy manure. Bioresour. Technol..

[31] Runge T., Zhang C. (2012). Two-Stage Acid-Catalyzed Conversion of Carbohydrates into Levulinic Acid. Ind. Eng. Chem. Res..

[32] Qin K., Yan Y., Zhang Y., Tang Y. (2016). Direct production of levulinic acid in high yield from cellulose: Joint effect of high ion strength and microwave field. RSC Adv..

[33] Shuai L., Pan X. (2012). Hydrolysis of cellulose by cellulase-mimetic solid catalyst. Energy Environ. Sci..

[34] Ding D., Wang J., Xi J., Liu X., Lu G., Wang Y. (2014). High-yield production of levulinic acid from cellulose and its upgrading to γ-valerolactone. Green Chem..

[35] Rout P.K., Nannaware A.D., Prakash O., Kalra A., Rajasekharan R. (2016). Synthesis of hydroxymethylfurfural from cellulose using green processes: A promising biochemical and biofuel feedstock. Chem. Eng. Sci..

[36] Huang Y.B., Fu Y. (2013). Hydrolysis of cellulose to glucose by solid acid catalysts. Green Chem..

[37] Hu L., Li Z., Wu Z., Lin L., Zhou S.Y. (2016). Catalytic hydrolysis of microcrystalline and rice straw-derived cellulose over a chlorine-doped magnetic carbonaceous solid acid. Ind. Crops Prod..

[38] Szabolcs A., Molnar M., Dibo G., Mika L.T. (2013). Microwave-assisted conversion of carbohydrates to levulinic acid: An essential step in biomass conversion. Green Chem..

[39] Morales-delaRosa S., Campos-Martin J.M., Fierro J.L.G. (2014). Optimization of the process of chemical hydrolysis of cellulose to glucose. Cellulose.

[40] Bai C.X., Zhu L.F., Shen F., Qi X.H. (2016). Black liquor-derived carbonaceous solid acid catalyst for the hydrolysis of pretreated rice straw in ionic liquid. Bioresour. Technol..

[41] Heidarian P., Behzad T., Karimi K. (2016). Isolation and characterization of bagasse cellulose nanofibrils by optimized sulfur-free chemical delignification. Wood Sci. Technol..

[42] Raspolli Galletti A.M., Antonetti C., de Luise V., Licursi D., Nassi N. (2012). Levulinic acid production from waste biomass. Bioresources.

